# The effective fraction isolated from Radix Astragali alleviates glucose intolerance, insulin resistance and hypertriglyceridemia in db/db diabetic mice through its anti-inflammatory activity

**DOI:** 10.1186/1743-7075-7-67

**Published:** 2010-08-24

**Authors:** Ruby LC Hoo, Janice YL Wong, CF Qiao, A Xu, HX Xu, Karen SL Lam

**Affiliations:** 1 Department of Medicine, LKS Faculty of Medicine, The University of Hong Kong, Hong Kong, China; 2 Chinese Medicine Laboratory, Hong Kong Jockey Club Institute of Chinese Medicine, Hong Kong, China; 3 Research Centre of Heart, Brain, Hormone, and Healthy Aging, LKS Faculty of Medicine, The University of Hong Kong, Hong Kong, China

## Abstract

**Background:**

Macrophage infiltration in adipose tissue together with the aberrant production of pro-inflammatory cytokines has been identified as the key link between obesity and its related metabolic disorders. This study aims to isolate bioactive ingredients from the traditional Chinese herb Radix Astragali (Huangqi) that alleviate obesity-induced metabolic damage through inhibiting inflammation.

**Methods:**

Active fraction (Rx) that inhibits pro-inflammatory cytokine production was identified from Radix Astragali by repeated bioactivity-guided high-throughput screening. Major constituents in Rx were identified by column chromatography followed by high-performance liquid chromatography (HPLC) and mass-spectrometry. Anti-diabetic activity of Rx was evaluated in db/db mice.

**Results:**

Treatment with Rx, which included calycosin-7-β-D-glucoside (0.9%), ononin (1.2%), calycosin (4.53%) and formononetin (1.1%), significantly reduced the secretion of pro-inflammatory cytokines (TNF-α, IL-6 and MCP-1) in human THP-1 macrophages and lipopolysaccharide (LPS)-induced activation of NF-κB in mouse RAW-Blue macrophages in a dose-dependent manner. Chronic administration of Rx in db/db obese mice markedly decreased the levels of both fed and fasting glucose, reduced serum triglyceride, and also alleviated insulin resistance and glucose intolerance when compared to vehicle-treated controls. The mRNA expression levels of inflammatory cell markers CD68 and F4/80, and cytokines MCP-1, TNF-α and IL-6 were significantly reduced in epididymal adipose tissue while the alternatively activated macrophage marker arginase I was markedly increased in the Rx-treated mice.

**Conclusion:**

These findings suggest that suppression of the inflammation pathways in macrophages represents a valid strategy for high-throughput screening of lead compounds with anti-diabetic and insulin sensitizing properties, and further support the etiological role of inflammation in the pathogenesis of obesity-related metabolic disorders.

## Background

Obesity is a major risk factor for a cluster of cardio-metabolic disorders including insulin resistance, fatty liver, dyslipidemia, type 2 diabetes and cardiovascular diseases [1,2]. Mounting evidence suggests that low-grade systemic chronic inflammation plays an important role in linking obesity to its associated pathologies.

Adipose tissue, originally regarded as an inert energy storage compartment, is now found to be an endocrine organ that secretes a large number of adipokines to regulate energy balance, food intake, lipid and glucose metabolism, insulin sensitivity and vascular tone [3]. It also plays a pivotal role in the development of systemic inflammation in obese subjects [4]. In obese humans and rodents, increased infiltration of activated macrophages or mast cells into adipose tissues is clearly evident [5]. Enlarged adipocytes, together with the infiltrated macrophages, act in a synergistic manner to cause aberrant production of pro-inflammatory molecules including inducible nitric oxide, cytokines such as tumor necrosis factor-alpha (TNF-α), interleukine-6 (IL-6) and the chemokine monocyte chemoattractant protein-1 (MCP-1). Both TNF-α and IL-6 can impede insulin sensitivity by triggering different key steps in the insulin signaling pathway [6-8]. Meanwhile, chronic inflammation impacts on the fat storage in adipose tissue, resulting in excess free fatty acid and triglycerides in the bloodstream, and the induction of insulin resistance in muscle and liver, in part via ectopic fat deposition in these tissues [1,9,10]. Weight loss has been shown to result in a reduction in macrophage accumulation and ameliorate the up-regulated inflammatory status in humans [11,12].

Radix Astragali, which is known as Huangqi in Chinese, is a flowering plant in the family Fabaceae. It is one of the 50 fundamental herbs used in traditional Chinese medicine and is traditionally used for the treatment of diabetes, wound healing [13] and strengthening the immune system [14,15]. Recently, a herbal formulation with Radix Astragali was shown to exert anti-hyperglycemic and anti-oxidant effect in the db/db diabetic mouse model [16]. In addition, our laboratory has demonstrated the anti-diabetic effect of two natural compounds from Radix Astragali, astragaloside II and isoastragaloside I, which can enhance the expression of adiponectin, an insulin-sensitizing adipokine [17]. However, the detailed mechanism underlying the anti-diabetic effects of Radix Astragali remains poorly understood.

Since the dysregulated production of cytokines and the activation of the inflammatory signaling pathways are closely associated with obesity-related metabolic diseases, we postulated that the beneficial metabolic effect of Radix Astragali may be mediated through anti-inflammatory actions. In this study, Radix Astragali was fractionated and its constituents were repeatedly screened for their activities in inhibiting pro-inflammatory cytokine production and lipopolysaccharide (LPS)-induced activation of the NF-κB signaling pathway in macrophages. The therapeutic potential of the selected active fraction Rx on the obesity-related metabolic disorders was validated in the db/db diabetic mice.

## Materials and methods

### Preparation of Bioactive Extracts from the Plant materials

Radix Astragali, the dried root of *Astragalus membranaceus *Bge. var.*mongholicus *(Bge.) Hsiao, was collected from Rui-Bao Good Agriculture Practice (GAP) base at Baotou City of Inner Mongolia in China in April 2008. A voucher specimen has been deposited in the Chinese Medicine Laboratory of Hong Kong Jockey Club Institute of Chinese Medicine. Radix Astragali was powdered and extracted with ethanol (20%, 50%, 80% and 95%) and water. The extract evaporated in vacuum and used for high-throughput screening. The effective fractions that inhibited pro-inflammatory cytokine production in macrophages was further extracted with various organic solvents (butanol and ethyl acetate) and their anti-inflammatory activities were further determined. To identify the major constituents in selected active fraction Rx, HPLC was performed in an Agilent 1100 system comprising of a quaternary pump, an online degasser, an auto-sampler, a column heater and a variable wavelength detector. Separation was achieved on a 4.6 × 250 mm, 5 μm particle, Alltima C_18 _reversed-phase analytical column. The mobile phase was acetonitrile and 0.1% aqueous formic acid; the amount of acetonitrile was changed linearly from 10 to 60% in 40 min. The flow rate was 1.0 ml/min and the wavelength was 254 nm. The proposed method for quantitative analysis of major constituents in the bioactive extracts was validated in terms of linearity, limits of detection and quantification, reproducibility and recovery. The identified active fraction was used for the treatment of db/db diabetic mice.

### Cell Culture and Macrophage Differentiation

Human THP-1 macrophage cells were maintained as sub-confluent cultures in RPMI-1640 supplemented with 10% fetal bovine serum and were induced for differentiation by incubating with 100 nM phorbol 12-myristate 13-acetate (PMA) for 3 days. After differentiation, medium was replaced with RPMI-1640 with 10% fetal bovine serum for 1 day before drug treatment. RAW-Blue cells (InvivoGen) were murine macrophages derived from RAW 264.7 cells. RAW-Blue cells were maintained as sub-confluent cultures in DMEM supplemented with 10% fetal bovine serum (Invitrogen). Cells were seeded in 24-well plate 1 day before drug treatment.

### Quantification of TNF-α, MCP-1 and IL-6 Production in Rx-treated Human THP-1 Macrophage Culture Medium

Conditioned THP-1 medium was collected after Rx (5 μg/ml or 10 μg/ml) or Rosiglitazone (10 μg/ml) treatment for 48 hours. The concentrations of TNF-α, MCP-1 and IL-6 were determined using in-house sandwich ELISA. The capture antibodies and detection antibodies of human TNF-α, MCP-1 and IL-6 were purchased from R & D systems (Minneapolis, MN).

### Detection and Quantification of LPS-induced Alkaline Phosphotase (SEAP) Activity in the Supernatants of RAW-Blue Cells

RAW-Blue cells were treated with LPS (100 ng/ml) alone or together with Rx (10 μg/ml or 20 μg/ml) for 24 or 48 hours. SEAP activity in the conditioned medium was determined using QUANTI-Blue medium (InvivoGen) following manufacturer's manual. Briefly, 20 μl samples were added to 200 μl of Quanti-Blue assay buffer (InvivoGen) and incubated at 37 C for 15 to 30 minutes. Absorbance was measured at 620 nm and fold change in SEAP activity in each sample compared to LPS-induced sample was calculated.

### Animal Studies

C57BL/KsJ db/db diabetic mice were propagated in the laboratory animal unit at University of Hong Kong. The mice were housed in a room under controlled temperature (23 ± 1 C), with free access to water and standard mouse chow. Mice at the age of 10 weeks were treated with either Rx (2 g/kg/day) or PBS with 4% Tween 80, as control, by daily oral gavage for 12 weeks. Physical parameters such as body weight and food intake of mice were measured weekly. Glucose tolerance test and insulin tolerance test were performed as previously described [18]. All of the experiments were conducted according to institutional guidelines for humane treatment of laboratory animals.

### Analysis of Serum Glucose, Free fatty acid, Triglyceride and Insulin Levels

Fed or fasting blood was collected from the tail-tip of mice. Trunk blood was collected by cardiac puncture under anesthesia before the mice were sacrificed. Serum glucose level was measured using the ACCU-CHEK Advantage II glucometer (Roche, USA). The levels of serum triglyceride and free fatty acid were determined using commercial assay kits Stanbio Liquicolor Triglycerides (Stanbio, USA) and Half micro test (Roche, USA), respectively. The level of insulin was measured by Ultra-sensitive mouse insulin ELISA kit (Mercodia, Sweden).

### Quantification of Inflammatory Marker Expression by Real-time Polymerase Chain Reaction (PCR)

Total RNA was extracted from mouse epididymal fat pads using Trizol reagent and was transcribed into cDNA with a Superscript first-strand cDNA synthesis system (Promega, Madison, WI, USA). The relative gene abundance was quantified by real time PCR using the assay on demand TaqMan primers and probes from Applied Biosystems (Foster City, CA) with the premade assay kits. The reactions were performed in an ABI 7000 sequence detection system.

### Statistical Analysis

Statistical analyses were performed using GraphPad Prism 3 software (San Diego, CA). Data are expressed as means ± S.E.M. Statistical significance was determined by one-way ANOVA and Dunnett's post hoc test. In all statistical comparisons, a P value < 0.05 was considered statistically significant.

## Results

### High Throughput Screening of the Active Fractions from Radix Astragali and HPLC Analysis of Four Main Constituents in selected fraction Rx

**Figure 1:**
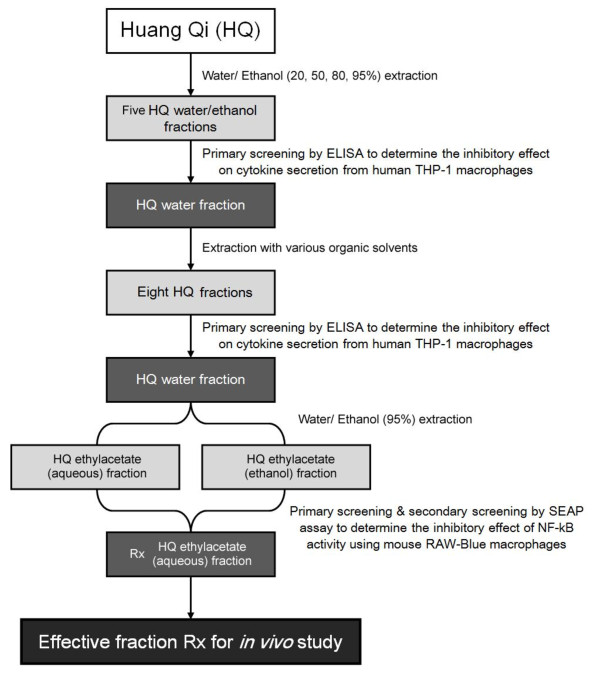
**The schematic diagram of the purification and identification of the active fractions from Radix Astragali**.

We selected about 20 ethanol and water extracts from three traditional Chinese herbs, Radix Astragali (Huang Qi), Rhizoma coptids Franch (Huang Lian) and Lonicerae japonica Thunb. (Jin Yin Hua) to screen for the compounds with anti-inflammatory properties. High-throughput screening, in which various fractions were incubated with human THP-1 for 24-48 hours followed by ELISA assays, allowed for the identification of components that reduced the basal cytokine secretion from macrophages. After repeatedly screening, we identified an active fraction (Rx) from Radix Astragali that reproducibly inhibited the pro-inflammatory cytokine secretion from macrophages. Its anti-inflammatory activity, as evidenced by inhibiting the NF-κB signaling pathway, was further confirmed by SEAP assay using mouse RAW-Blue cell system. The schematic diagram of the purification and identification of active fraction Rx from Radix Astragali was shown in figure 1.

**Figure 2:**
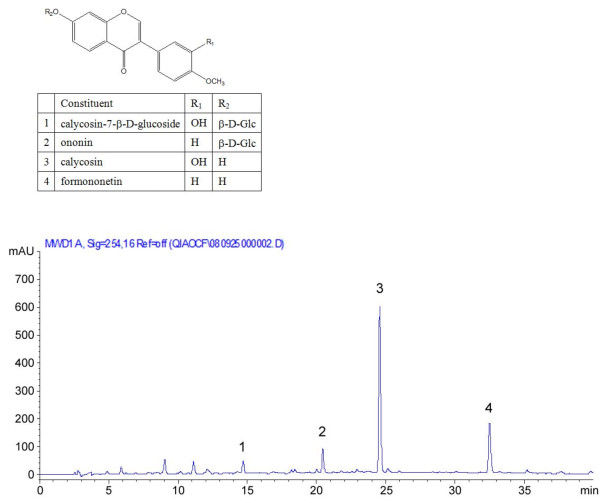
**The chemical structures of the four major constituents in the Rx and HPLC chromatogram of the Rx fraction**.

The HPLC analysis results showed that Rx contained calycosin-7-β-D-glucoside (0.9%), ononin (1.2%), calycosin (4.53%) and formononetin (1.1%), respectively. The representative HPLC chromatogram was shown in figure 2.

### Effect of the Fraction Rx on Secretion of Pro-inflammatory Cytokine and LPS-induced NF-κB Activation in Macrophages

**Figure 3:**
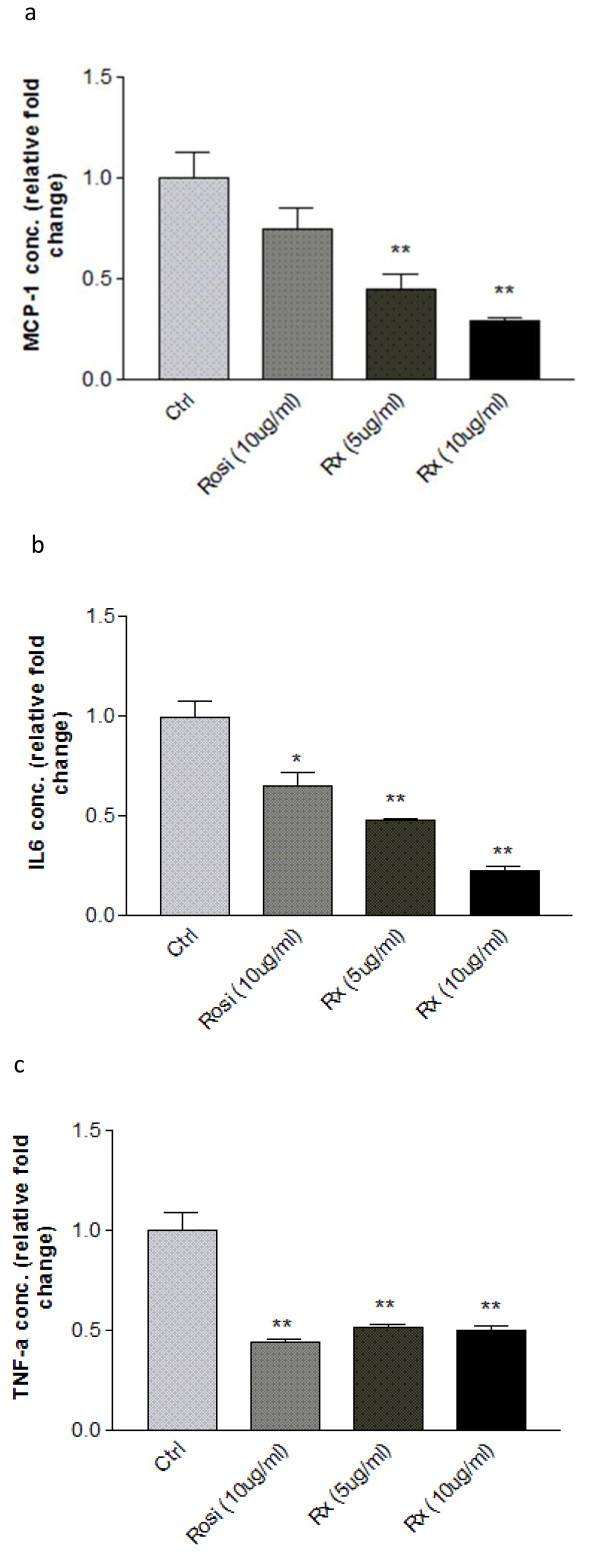
**Rx significantly reduced the secretion of cytokines in human THP-1 macrophages**. Panels show (a) MCP-1, (b) IL-6 and (c) TNF-α concentrations in human THP-1 macrophage conditioned medium after incubation with Rosiglitazone (10 μg/ml) or Rx (5 μg/ml or 10 μg/ml) for 48 hours. Each bar represents the relative mean fold change ± SEM (n = 6). Data were statistically analyzed using the one-way ANOVA with Dunnette's post hoc test. * P < 0.05; ** P < 0.01.

**Figure 4:**
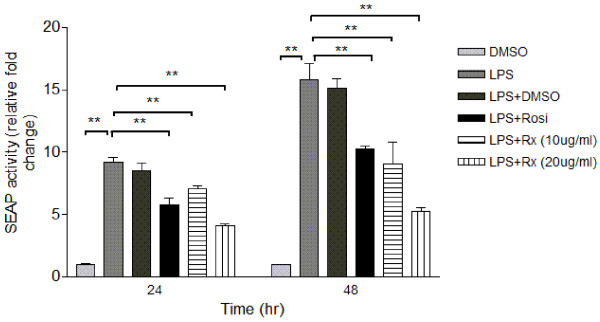
**Rx significantly inhibited LPS-induced NF-κB activity (as indicated by SEAP activity) in mouse RAW-Blue macrophages**. Different dosages of Rx (10 μg/ml or 20 μg/ml) or Rosiglitazone (10 μg/ml) together with 100 ng/ml LPS were incubated with RAW-Blue cells for 24 hours or 48 hours. DMSO was used as the vehicle control. The SEAP activities in the conditioned medium of mouse RAW-Blue cells were measured using QUANTI-Blue assay as described in Methods. Each bar represents the relative mean fold change ± SEM (n = 6). Data were statistically analyzed using one-way ANOVA with Dunnette's post hoc test. ** P < 0.01.

Two macrophage cell systems, human THP-1 macrophages and mouse RAW-Blue macrophages, were employed in the high-throughput screening platform to determine the effects of the active fractions. Treatment with the selected active fraction Rx caused a drastic reduction in secretion of pro-inflammatory cytokines including MCP-1 and IL-6 in the conditioned medium of THP-1 macrophages, in a dose-dependent manner (figure 3a, b). The inhibitory effect of Rx (5 μg/ml and 10 μg/ml) on TNF-α secretion was comparable to that of Rosiglitazone (10 μg/ml) (figure 3c). On the other hand, Rx had a more effective inhibitory effect than Rosiglitazone on the secretion of MCP-1 and IL-6 (figure 3a, b). The anti-inflammatory effect of Rx was further confirmed by secondary screening using mouse RAW-Blue cells. RAW-Blue cells are derived from RAW-264.7 macrophages that stably express a secreted embroyonic alkaline phosphotase (SEAP) gene which can be induced by NF-κB or AP-1 transcription factors. Treatment of RAW-Blue cells with Rx inhibited the LPS-induced NF-κB activity in a time- and dose-dependent manner as indicated by the reduced SEAP activity (figure 4). The effect of Rx was also comparable to Rosiglitazone (10 μg/ml). Rx was then manufactured in a large quantity for the *in vivo *study.

### Effect of the Fraction Rx on Metabolic Parameters of db/db Diabetic Mice

**Figure 5:**
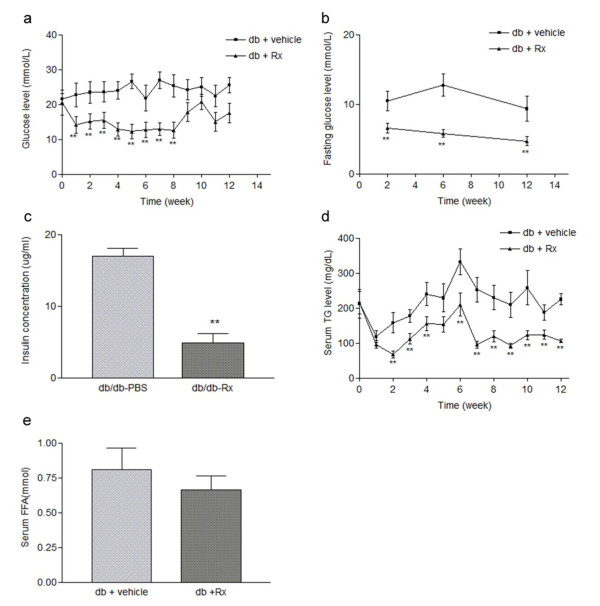
**Rx decreased both fed and fasting blood glucose levels and alleviated hypertriglyceridemia in db/db diabetic mice**. Male db/db diabetic mice were treated with vehicle or Rx (2 g/kg/day) for 12 weeks by oral gavage. (a) The weekly profile of fed glucose levels. (b) The fasting glucose levels at week 2, 6 and 12 measured after 16 hours of starvation. (c) Fasting serum insulin concentration after 12 weeks of Rx treatment. (d) The weekly profile of fed triglyceride level. (e) Serum free fatty acid concentration after 12 weeks of Rx treatment. Data were statistically analyzed using one-way ANOVA with Dunnette's post hoc test. Data are presented as mean ± S.E.M. (n = 5-6). ** P < 0.01.

Having established the action of Rx on inhibiting cytokine secretion from macrophages, we next validated the therapeutic potential of the active fraction Rx in the db/db mice, a well-established genetic obese model with typical symptoms of type 2 diabetes. Male db/db mice were administrated with Rx fraction (2 g/kg/day dissolved in 4% Tween 80 in PBS) or vehicle by daily oral gavage for a period of 12 weeks. The effects of Rx on insulin sensitivity and glucose metabolism were investigated. Rx treatment markedly improved the glycemic control in db/db mice. Within 3 weeks of treatment, the fed glucose levels were significantly reduced and this effect was persistent throughout the treatment period (until week 7). In order to determine whether the therapeutic effect of Rx would persist after drug withdrawal, we stopped the administration of Rx for 2 weeks (week 8-10) (figure 5a). The fed blood glucose level remained lowered for one week (week 8) after drug withdrawal and then gradually increased from week 8 to week 10, but was reduced again when Rx was re-administrated to the mice (week 11-12). Similarly, the fasting glucose levels and serum insulin concentrations were significantly reduced in Rx-treated db/db mice (figure 5b,c). Rx treatment also dramatically improved the hypertriglyceridemia in db/db mice within 2 weeks of treatment, with the effect lasting throughout the treatment period and persisting through the two weeks of drug withdrawal (figure 5d). A trend of decreased serum free fatty acid (FFA) was also observed (figure 5e). These data suggested that Rx treatment could improve the glycemic and lipid control in db/db mice.

**Figure 6:**
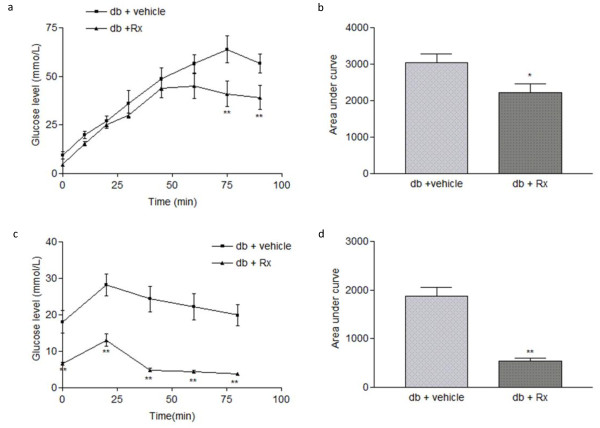
**Rx treatment alleviated glucose intolerance and insulin resistance in db/db diabetic mice**. Male db/db diabetic mice were treated with vehicle or Rx (2 g/kg/day) for 12 weeks by oral gavage. (a) Glucose tolerance test (GTT) conducted at week 12 after treatment. (b) Glucose tolerance test expressed as area under curve. (c) Insulin tolerance test (ITT) conducted at week 12 after treatment. (d) Insulin tolerance test expressed as area under curve. All mice were fasted for 16 hours or 6 hours before GTT and ITT were carried out, respectively. Data were statistically analyzed using one-way ANOVA with Dunnette's post hoc test. Data are presented as mean ± S.E.M. (n = 5-6). * P < 0.05; ** P < 0.01.

Glucose tolerance test and insulin tolerance test were performed to examine the systemic glucose metabolism and insulin sensitivity in db/db mice. Rx-treated db/db mice displayed a more efficient clearance of systemic glucose level and had a significant increase in insulin sensitivity than the vehicle-treated db/db mice (figure 6a, b, c, d).

### Effect of Rx Treatment on the Adipose Tissue of the db/db Diabetic Mice

**Figure 7:**
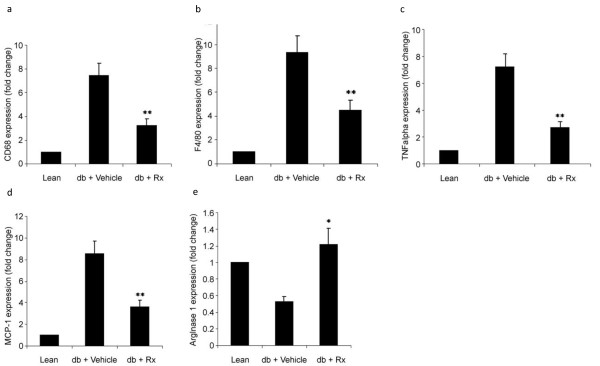
**Rx inhibited the inflammatory state in adipose tissue of db/db diabetic mice**. Panels show the relative mRNA levels of the inflammatory cell markers (a) CD68 and (b) F4/80, pro-inflammatory cytokines (c) TNF-α and (d) MCP-1 and alternatively activated macrophage marker (e) arginase I in adipose tissue of vehicle- or Rx-treated db/db diabetic mice and lean control. Data were statistically analyzed using one-way ANOVA with Dunnette's test. Data are presented as relative mean fold change ± S.E.M. (n = 5-6). * P < 0.05; ** P < 0.01.

Quantitative real time PCR analysis showed that chronic treatment with Rx resulted in a significant reduction in the mRNA levels of two macrophage markers, CD 68 and F4/80, in the epididymal adipose tissue of db/db mice when compared with the vehicle-treated mice (figure 7a, b). The mRNA levels of the pro-inflammatory cytokines, TNF-α and MCP-1, were also markedly reduced in the adipose tissue of the Rx-treated mice (figure 7c, d). In contrast, the mRNA levels of the anti-inflammatory or "alternatively activated" macrophages arginase I gene, was significantly increased in the Rx-treated mice (figure 7e). These findings suggest that Rx treatment reduced inflammation status in the adipose tissue.

### Physical Parameters in Experimental Animals

**Figure 8:**
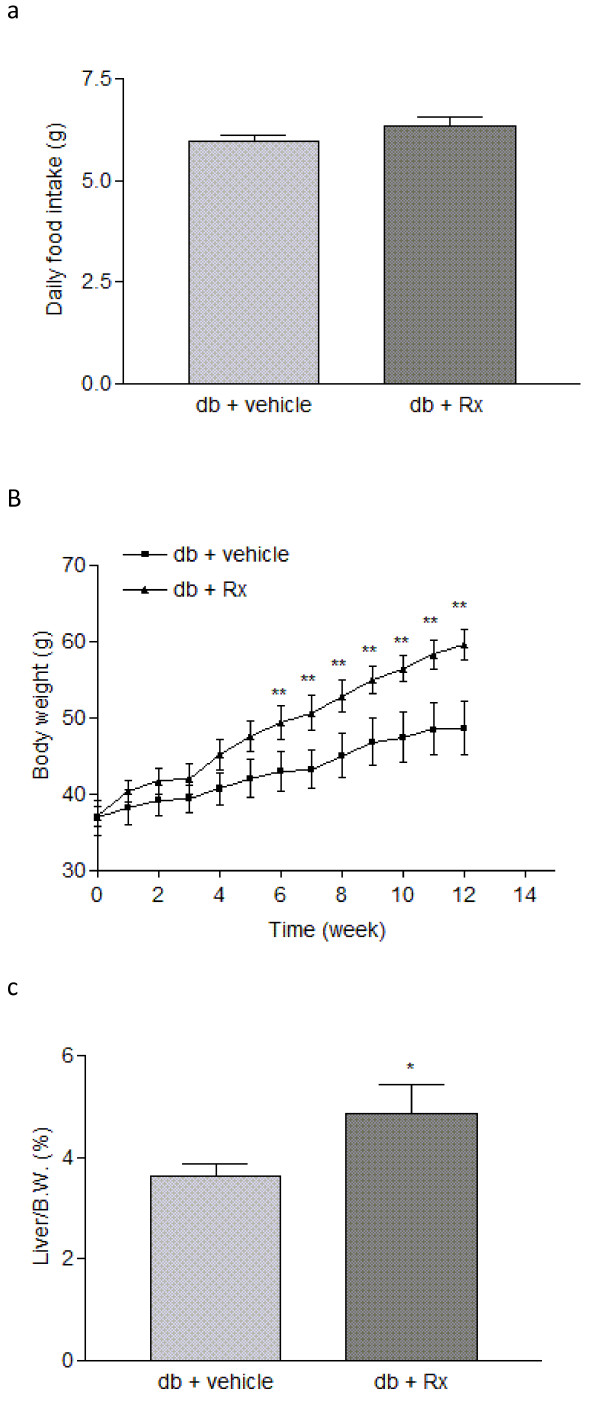
**Effects of Rx on the basic metabolic parameters of db/db diabetic mice**. Panels show (a) daily food intake, (b) body weight and (c) liver weight of the treated db/db diabetic mice. Data were statistically analyzed using one-way ANOVA with Dunnette's test. Data are presented as mean ± S.E.M. (n = 5-6). * P < 0.05; ** P < 0.01.

**Figure 9:**
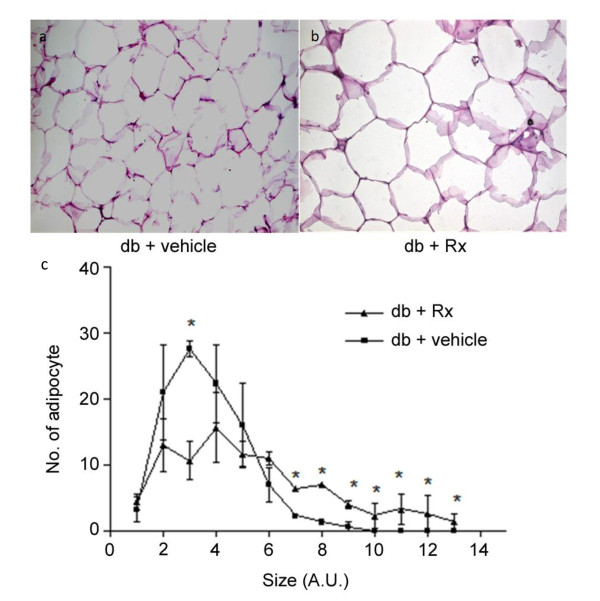
**Rx treatment increased the adipocyte size of db/db diabetic mice**. Panels show the representative images of hemotoxylin and eosin-staining of epididymal adipose tissue of (a) db/db mice treated with vehicle and (b) db/db mice treated with Rx (2 g/kg/day). (c) Quantification of the adipocyte size. Data were statistically analyzed using one-way ANOVA with Dunnette's post hoc test. Data are presented as mean ± S.E.M. (n = 5-6). * P < 0.05.

**Figure 10:**
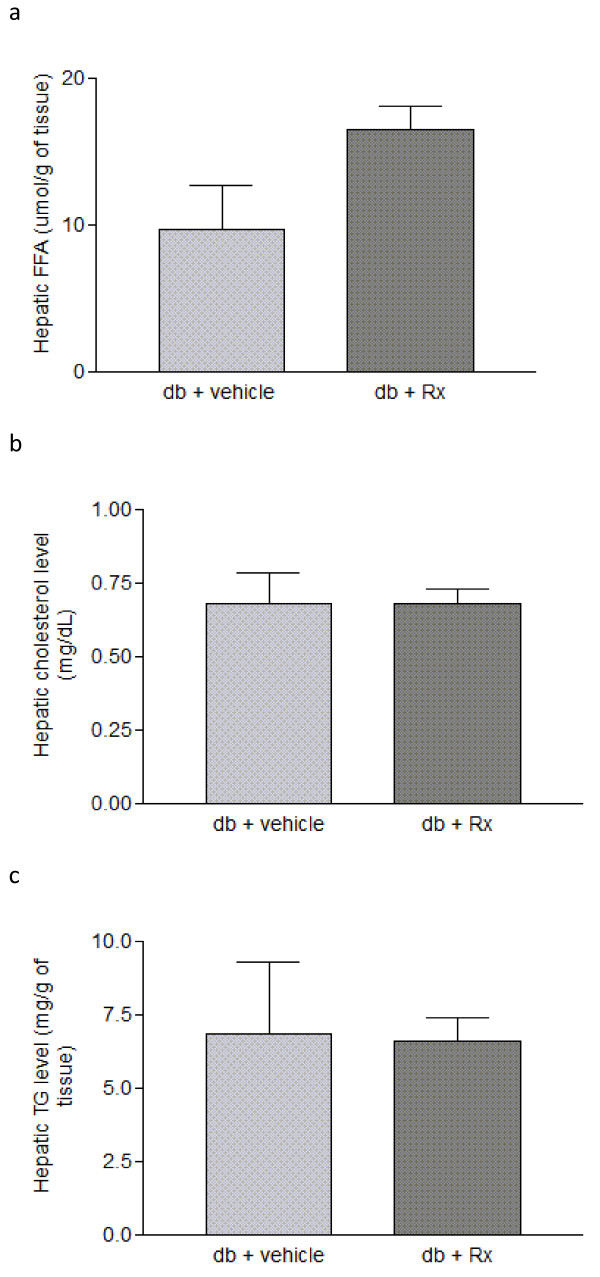
**Rx treatment did not affect the hepatic lipid profile of db/db diabetic mice**. Panels show the hepatic (a) free fatty acid (FFA), (b) cholesterol and (c) triglyceride concentrations of db/db diabetic mice. Data were statistically analyzed using one-way ANOVA with Dunnette's test. Data are presented as mean ± S.E.M. (n = 5-6).

Treatment of db/db mice with Rx did not affect daily food uptake (figure 8a). However, there was an increased body weight in the Rx-treated group (figure 8b). This may be due to the increased liver weight (figure 8c) and adipocyte size (figure 9). Biochemical analysis on the hepatic lipid profile showed that there were no significant changes in hepatic triglyceride, cholesterol and free fatty acid in the Rx treated mice (figure 10) when compared with vehicle-treated mice.

## Discussion

The prevalence of diabetes has become a major public health concern worldwide and is known to be closely associated with obesity. Cumulating evidence suggests that chronic inflammation in white adipose tissue (WAT), which is characterized by infiltrated macrophages and aberrant production of pro-inflammatory cytokines, plays an essential role in linking obesity with diabetes and its complications [18,19]. Insulin resistance precedes impaired glucose tolerance and the onset of type 2 diabetes. In obese subjects, infiltrated macrophages together with enlarged adipocytes secrete numerous pro-inflammatory mediators such as IL-6 and TNF-α, which contribute to insulin resistance [6,20]. The cytokine-stimulated adipocytes secrete chemokines such as MCP-1 which further enhance the recruitment of macrophages into the adipose tissue, hence amplifying the inflammatory response [21-23].

Though there are existing synthetic anti-diabetic drugs that possess anti-inflammatory properties, such as the thiazolidenediones (TZDs), an increasing number of studies has documented their undesirable effects including the increased risk for heart failure and bone fractures [24]. Therefore, it is important to identify new anti-diabetic drug with anti-inflammatory actions that are associated with a better safety profile. With the increased popularity of herbal medicine, many Chinese herbs are now frequently used as the natural and presumably safer sources for drug discovery. Metformin, a widely used anti-diabetic drug, was identified from the herb French lilac (Galega officinalis) [25,26]. Berberine, identified from the traditional herb Hydrastis canadensis or Coptis chinensis which has been used for treating diabetes in China for more than 1400 years, has recently been shown to have glucose lowering, insulin sensitizing and incretin actions [27-29].

In the present study, we have developed a high-throughput screening platform, for selection of active fractions from traditional Chinese herbs that possess anti-inflammatory properties, which is based on the ability of the fraction to inhibit cytokine production in macrophages. An effective fraction Rx from Radix Astragali was identified after repeated screening and its anti-diabetic effect was confirmed in the db/db mouse model.

Radix Astragali is an edible herb that has been used as one of the primary tonic herbs in China for many centuries [30]. Recently, various studies on the effects of Radix Astragali on obesity-related metabolic disorders have been carried out [31,32]. The extracts of Radix Astragali have been shown to be anti-inflammatory [33], hepatoprotective [34], cardioprotective [35], neuroprotective [36,37] and anti-diabetic [38]. The use of Radix Astragali to treat diabetic symptoms (Xiao Ke, or wasting thirst syndrome, in Chinese medicine) has been well documented in the Compendium of Materia Medica (Ben Cao Gang Mu) during the Ming Dynasty (from 1368-1644 AD). Notably, Radix Astragali is now being used as a major ingredient in six of the seven anti-diabetic herb formulae approved by the State Drug Administration in China [38].

Radix Astragali contains various isoflavones and isoflavonoids including formononetin, calycosin and ononin, and many saponins including astragaloside I, astragaloside II, astragalosie VI and also acetylastragaloside I [39]. Our laboratory has demonstrated that astragaloside II and isoastragaloside I from Radix Astragali alleviate insulin resistance, glucose intolerance and hyperglycemia by increasing the secretion of the insulin sensitizing hormone, adiponectin from adipocytes [17]. The effective fraction Rx is a mixture of 4 isoflavonoids including calycosin-7-β-D-glucoside (0.9%), ononin (1.2%), calycosin (4.53%) and formononetin (1.1%). Formononetin and calycosin have been shown to be activators of peroxisome proliferator-activated receptors (PPARα and PPAR-γ) [40], the major therapeutic targets of the fibrate group of lipid-lowering drugs the TZDs, respectively. It should be noted that PPAR-γ is highly expressed in the adipose tissue where it regulates insulin sensitivity, adipocyte differentiation and lipid storage, and is responsible for regulating the alternative activation of macrophages [41]. Formononetin and calycosin have also been reported to potentiate the anti-hyperglycemic action of fangchinoline [42] and inhibit the LPS-induced production of TNF-α, nitric oxide and superoxide in mesencephalic neuron glia culture [43]. On the other hand, calycosin has a protective effect in endothelial cells against hypoxia-induced barrier impairment [44], while calycosin-7-O-β-D-glucoside and ononin have been reported to be free radicals scavenging anti-oxidants [45]. All these studies have suggested that the four major components of Rx would have potential anti-inflammatory, glucose-lowering and anti-oxidant effects which may contribute to improving the insulin resistance, glycemic and lipid control in the Rx-treated db/db mice. However, there is no evidence suggests that these components are bioactive ingredients. Further experiments are needed to verify it.

The Food and Drugs Administration (FDA) is now recommending a mixture of multiple active ingredients rather than a single active compound for herbal alternative medicine. This is due to the fact that the constituents can work together to enhance the effectiveness and potentiate a synergistic effect, so that the dosage and toxicity can be reduced [46].

Our present study has demonstrated that Rx significantly reduced the plasma glucose and triglyceride levels with the effect persisting throughout the experimental period (figure 5a, b, d). Rx treatment also reduced the expression of macrophage markers and pro-inflammatory cytokines in the adipose tissue, indicating an ameliorating effect on chronic inflammation in the db/db diabetic mice. The increased arginase I expression implied an increased alternative activation of macrophages in the adipose tissue, which could lead to the secretion of the anti-inflammatory cytokines IL-10 and IL-8 [47], indicating that Rx treatment may lead to polarization of resident macrophage towards the alternative state. Though increased body weight in db/db mice has been observed after Rx treatment, this is also a common effect of the well-established PPAR-γ agonist, TZDs, in spite of their anti-diabetic effect.

In summary, the present study raised the possibility that measurement of cytokine production level in macrophages is a viable activity-guided high-throughput screening method to search for the lead compounds with anti-diabetic potential. Using this platform an effective fraction Rx was identified from the traditional Chinese herbal medicine Radix Astragali and shown to demonstrate anti-diabetic and lipid-lowering effects, at least in part via the suppression of inflammation in adipose tissue.

## Competing interests

The authors declare that they have no competing interests.

## Authors' contributions

RH carried out the drug screening, animal study, data analysis, designed and coordinated the study and drafted the manuscript. JW carried out drug screening and animal work. CQ carried out the purification and identification of the active fraction. AX participated in design the study and helped to draft the manuscript. HX designed the study and helped in data analysis on the active fraction purification. KL conceived the study. All authors read and approved the final manuscript.
